# Restructuring and serving web-accessible streamflow data from the NOAA National Water Model historic simulations

**DOI:** 10.1038/s41597-023-02316-7

**Published:** 2023-10-20

**Authors:** J. Michael Johnson, David L. Blodgett, Keith C. Clarke, Jon Pollak

**Affiliations:** 1 Lynker, Fort Collins, CO USA; 2 University of California, Santa Barbara, USA; 3 U.S. Geological Survey, Reston, USA; 4 Consortium of Universities for the Advancement of Hydrologic Science, Inc, Cambridge, USA

**Keywords:** Hydrology, Environmental impact

## Abstract

In 2016, the National Oceanic and Atmospheric Administration deployed the first iteration of an operational National Water Model (NWM) to forecast the water cycle in the continental United States. With many versions, an hourly, multi-decadal historic simulation is made available to the public. In all released to date, the files containing simulated streamflow contain a snapshot of model conditions across the entire domain for a single timestep which makes accessing  time series a technical and resource-intensive challenge. In the most recent release, extracting a complete streamflow time series for a single location requires managing 367,920 files (~16.2 TB). In this work we describe a reproducible process for restructuring a sequential set of NWM steamflow files for efficient time series access and provide restructured datasets for versions 1.2 (1993–2018), 2.0 (1993–2020), and 2.1 (1979–2022). These datasets have been made accessible via an OPeNDAP enabled THREDDS data server for public use and a brief analysis highlights the latest version of the model should not be assumed best for all locations. Lastly we describe an R package that expedites data retrieval with examples for multiple use-cases.

## Background & Summary

Streamflow records provide information for a range of people including emergency responders, water managers, environmental and transportation agencies, researchers, utility companies, and consulting firms^1–3^. Specific needs might include short- and long-range planning^4,5^, warning about floods and droughts^6–8^; managing water rights, regulating and monitoring environmental impacts^9,10^; operating waterways for commerce; and designing flood frequency curves^11,12^.

Despite the vast utility of streamflow records, there is a divergence between where water exists and where it is measured even in densely gaged countries^13,14^. Over the last few decades, continental to global scale hyper-resolution hydrologic prediction has been dubbed a “grand challenge” within the hydrology community to address this shortcoming^15–17^. Although many have praised the scientific and societal advantages these massive models offer, there is a steep set of technical, conceptual, and practical hurdles^18,19^.

In the United States, the National Oceanic and Atmospheric Administration (NOAA) undertook the development of an operational, high-resolution model as part of the strategic mission of building a ‘weather-ready’ nation^20^. In 2016, version 1.0 of the National Water Model (NWM) was put into operation^21,22^ to provide the first-ever continental United States-wide modelling capability using real-time weather forecasts, a high resolution (1 km^2^) land surface model^23,24^, and a multi-resolution surface routing model^25,26^. Shortly following the initial release, version 1.2 expanded the calibration basins from 40 to more than 1,000 and improved parameter regionalization^27^. Version 2.0 expanded the NWM domain further to include Hawaii and added new configurations (medium-range ensemble); out-of-bank compound channels parameterizations; and improved snow physics. Many land surface and hydrologic parameters were further refined by expanding the calibration basin set to approximately 1,400 basins^28^. Version 2.1 saw the expansion to Puerto Rico, the U.S. Virgin Islands, and the Great Lakes region and included improved reservoir treatment and modifications to the model’s snow and runoff parameters^29^. This version also began using the Analysis of Record for Calibration^30,31^ (AORC) dataset to enhance calibration and improve the estimation and regionalization of hydrologic parameters^32^.

 Although the timesteps, horizons, ensemble members, configurations, and file names have evolved with each release, the model has consistently produced 1 km^2^ gridded files of land surface and forcing states, a 250 m^2^ gridded file of the terrain conditions (ponded water), and point files containing the stream routing and reservoir variables for the entire domain. Starting with version 1.2, a multi-decadal, hourly, historical simulation has been released with most versions providing a resource for better understanding the NWM and the earth system it simulates. Versions 1.2 and 2.0 of the historical simulations used the NLDAS/NARR forcings^33–36^ whereas v2.1 used the AORC dataset. These historic simulations provide an unprecedented resource that can spur research and understanding about the model, its evolution, and its applications^37–40^, and can help better understand what improvements were made version to version.

Despite all historic simulation data being available on on Amazon Web Services (AWS) Registry of Open Data^41^, the data structure can be hard to use for specific use cases. In the case of long-term streamflow records there are three primary challenges to overcome. First, the point files contain a snapshot of conditions for a given domain (CONUS) and timestep (1 hour). As a result, extracting a single time series for a location of interest would require managing anywhere from 4 (for v1.2) to 16 (v21.1) terabytes of data.

Second, the hourly point files are structured to prioritize space-based, rather than time-based subsetting even when concatenated with common open-source tools. Although this can be a typical design pattern for spatial grids, it is limiting when trying to extract time oriented data.

Lastly, the point datasets index 1D variables using a non-spatial, non-sequential, identifier (feature_id) that is adopted from the common identifiers (COMID) associated with stream reaches in the NHDPlusV2^42^. This requires users to first find the identifiers of interest, then use the position of that identifier in the dataset to extract the needed records. In the operational data, there is no spatial information associated with these feature_ids and NOAA states that “due to storage space limitations, the latitude and longitude of each point are stored in an external Esri file geodatabase…”^30^. In the historic data (v2.0 and v2.1) coordinate data were added to the channel files at the expense of increasing the average file size from 6.6 MB to 47.6 MB. While adding the capacity for pseudo spatial subset (given the streamflow variable is not indexed to theses coordinate dimensions), it exacerbates the amount of data that needs to be managed. 

When looking at common use patterns for optimal streamflow dissemination we focused on the design of the U.S. Geologic Survey (USGS) National Water Information System (NWIS) which delivers data by site through time, rather than by time across all sites^43,44^. During the 2017 water year alone more than 640 million requests for streamflow data were fulfilled by NWIS, with 98% being fulfilled by webservices^2^. Thus, we believe NWIS can serve as a guide for an optimized streamflow dataset located in a centralized web-accessible resource.

In this data descriptor we highlight the approach developed for restructuring NWM point files for time-based access and restructure the streamflow records from v1.2, v2.0, and v2.1. The data are served by an OPeNDAP-enabled THREDDS data server, and we illustrate how data can be discovered programmatically using combinations of publicly available tools and a corresponding R package called nwmTools.

## Methods

The key technologies used to aggregate and reshape the NWM channel output files include the Network Common Data Form (NetCDF) data model^45^ (https://docs.unidata.ucar.edu/netcdf-c/current/netcdf_data_model.html) and a THREDDS data server configured with OPeNDAP access^46^. NetCDF files are a common, platform independent, and self-describing data format used to store multi-dimensional, array-based variables. The explicit entities in any NetCDF file include the *dimensions*, *variables*, and *data*^47^.

*Dimensions* are one-dimensional arrays with a name and size. They may represent a physical property, like time and latitude, or abstract values like the unique identifiers in the channel files. Record dimensions are defined with a length of “unlimited,” allowing them to grow with data being written to the file, or through the concatenation of multiple files. Critically, non-record dimensions cannot grow in this way.

*Variables* are defined by a name and shape determined by an ordered set of dimensions. For example, *rainfall(latitude, longitude, time*) is a three-dimensional variable with an X,Y, and T dimension. For any variable, the last dimension in the above syntax varies the fastest, while the first varies the slowest. The implications of this are that the shape of a variable directly impacts the performance for a specific use case.

In the NWM point files, streamflow is shaped as *streamflow(feature_id)* with an ‘‘unlimited’’ global record time dimension. If two files were concatenated along the time dimension, the variable would become a 2D variable *streamflow(time, feature_id)* which prioritizes extraction along the feature_id. Changing the order of dimensions requires pivoting the variable array to achieve *streamflow(feature_id, time)* which again can be quite intensive for large arrays.

With respect to sharing data, its long been understood that many NetCDF datasets are too big to be efficiently shared via regular upload and download. This has spurred the development of remote access technologies. One of these options is a THREDDS Data Server (TDS) which uses the NetCDF-Java/CDM library to read multidimensional data into a Common Data Model (CDM)^45,46,48^. Once in a TDS, any CDM resources can be accessed using a uniform resource locator (URL). THREDDS catalogues allow users to navigate what data are available in a browser and can leverage the NetCDF Markup Language (NcML) to modify and aggregate multiple CDM datasets into an entity that acts as a single resource.

An integrated TDS server can also provide OPeNDAP access which extends the HTTP protocol to allow CDM subsets to be requested via URL. Subsets are defined by appending constraint expressions to the CDM URL in the form of ?*variable[X:Y:Z]*. This expression will return the data from the named variable array at index X to index Z with an interval of Y along the first dimension. OPeNDAP arrays are zero indexed, thus *?time[3:1:3]* would return the fourth value in the 1D time dimension and *?streamflow[0:1:0][0:1:9]* would return the first ten streamflow records for the first *feature_id* in the streamflow array organized as (feature_id, time). OPeNDAP requests can be submitted through any Data Access Protocol (DAP) client (including a standard web browser) and any tool that uses the C-based NetCDF Application Programming Interface (API) acts as a DAP client making OPeNDAP available in most common programming languages.

With this background, we can leverage these technologies to develop a performant, time series-oriented channel output file. To do this, a set of sequential NWM files must be identified and an iterative process applied in which: (1) all variables except feature_id, time, and variable of interest (e.g. streamflow) are dropped; (2) the scale factor and offsets are removed; (3) the dimensions of the variable are redefined to be *streamflow*[*time, feature_id*]; and (4) the time dimension is set to the record dimension. Once complete, all files can be merged along the time dimension creating a single 2D variable. To prioritize time series access, the variable is reshaped as *streamflow*(*feature_id*, *time*) after which the scale factor and offset are reintroduced. Optionally the file can be rechunked and compressed for performance. Table 1 provides pseudo-NCO code of this process and a plain language explanation.

**Table 1:** The process for concatenating and pivoting a collection of NWM channel files leveraging the NCO^61^ tool set. Panel A outlines the user-defined variables which are bolded throughout the table. Panel B describes the steps in terms of NCO pseudo code. Panel C provides a plain language summary of the steps demonstrated in panel B along with references to the appropriate NCO utilities.

**A. User0defined variables**		
**FILELIST** = collection of sequential NetCDF model output files		
**OUTFILE** = desired output file to create (path)		
**VAR** = variable name (e.g. streamflow)		
B. Concatenate & Pivoting Operations Step-by-Step		
1	ITERATE (Over *i*)	*ncks -O -4 -L 1 --cnk_plc* = *all --cnk_map* = *dmn -C -v feature_id,time*, **VAR FILELIST[i] FILELIST[i]**
	| |	*ncatted -O -a “scale_factor*,**VAR***,d,,” -a “add_offset*,**VAR***,d,,”‘* **FILELIST[i] FILELIST[i]**
	| |	*ncap2 -O -s VAR [time,feature_id]* = ***VAR*** **FILELIST[i] FILELIST[i]**
	| STOP	*ncks -O --mk_rec_dmn time* **FILELIST[i] FILELIST[i]**
2		*ncrcat -O -6* **FILELIST OUTFILE**
3		*ncpdq -O -a feature_id,time* **OUTFILE OUTFILE**
4		*ncatted -h -O -a “scale_factor*,**VAR***,o,f,0.01”* **OUTFILE OUTFILE**
5		*ncks -O --cnk_plc = g2d --cnk_dmn feature_id,10000 --cnk_dmn time*,#**FILELIST OUTFILE OUTFILE**
6		*ncks -4 -L 3 -O* **OUTFILE OUTFILE**
**C. Description**		
**Plain language operations:**		
1. Start Iterating over files and for each:		
a. Extract the desired **VAR**, feature_id, and time 1D variables		
b. Delete (d) the scale factor (double) and add_offset(double) attribute in each file		
c. Reshape the 1D **VAR** to be [time,feature_id]		
d. Set the time dimension to the record dimension allowing it to ‘grow’		
Stop Iteration		
2. Concatenate files across the time dimension		
3. Re-shape **VAR** such that the dimensions are [feature_id, time]		
4. Overwrite (o) the scale_factor of **VAR** to be a float (f) of value 0.01		
4. Rechunk the time dimension (#**FILELIST**) and feature_id dimension (10,000)		
4. Compress the file		
**NCO utilities:**		
1. https://linux.die.net/man/1/ncks		
2. https://linux.die.net/man/1/ncatted		
3. https://linux.die.net/man/1/ncap2		
4. https://linux.die.net/man/1/ncrcat		
5. https://linux.die.net/man/1/ncpdq		

Operational NWM outputs are publicly available for a 48-hour rolling window on the NOAA Operational Model Archive and Distribution System (NOMADs)^49^. Since the release of v2.0 (2018), these have been archived in Google Cloud Platform (GCP) with a minimal lag. The method proposed here works on any sequential set of NWM data from any of these sources which have small to modest file lists that can be downloaded, merged, and reshaped on local hardware and memory. Although this responsibility could reside with an authoritative organization (either natively in the model, or as a post-processing step), it is feasible for users to execute themselves as new forecasts become available.

In contrast, the historical simulations on the AWS Registrary of Open Data^41^ constitute a much larger file set (up to 16 TB for v2.1) which require either massive hardware, or a divide and conquer approach to execute. We elected the latter approach and each month in the historic record was treated as a unique file list (n = ~744 hourly files). Steps 1 (file processing) and 2 (concatenation) were completed to create a set of monthly files. Each monthly file was split into 273 new files containing ~10,000 *feature_ids* each. The respective subsets from each month were then merged with their counterparts along the time dimension producing 273 files with the entire s*treamflow[time, feature_id]* variable for ~10,000 feature_ids. This space and time divide-and-conquer approach was designed to optimize speed while allowing the processing to remain in memory.

The files were then joined with an NcML aggregation and pivoted within a local THREDDS workspace using the *nccopy* utility (see code at https://linux.die.net/man/1/nccopy)^50^. The result is 273 time-optimized files that act as a single logical entity via a NcML file.

## Data Records

The complete dataset for the three version of the NWM are distributed across servers. The composite resource for all versions has been documented in HydroShare^51^. Version 1.2 and 2.0 are available on the HydroShare partition of the Renaissance Computing Institute at the University of North Carolina at Chapel Hill, and version 2.1 is hosted by the USGS Center for Integrated Data Analytics  (see Table 2 for key web addresses).

Each version of the dataset is structured in the same way regardless of server and includes a top level THREDDS directory containing a subdirectory of 273 time-optimized NetCDF files and a NcML file that allows that directory to act as a single resource. Table 2 documents the root path to each version along with the naming convention of the NetCDF and NcML files. Each individual file (and resulting NcML file) has a 1D latitude, longitude, time, and feature_id variable along with a 2D streamflow array. The content and associated metadata can be viewed at the NcML catalog page and can be accessed by any DAP client.

**Table 2:** This table lists the THREDDS catalog root directory and file naming convention for each version of the data. The XXX represents a three-digit number, padded with leading zeroes ranging from 1 to 273.

Version	Root	NetCDF Naming	NcML Name
1.2	https://thredds.hydroshare.org/thredds/catalog/nwm/retrospective/		
		*nwm_retro_full/nwm_retro_XXX.nc*	*nwm_retro_full.ncml*
2.0	https://thredds.hydroshare.org/thredds/catalog/nwm/retrospective/		
		*nwm_v2_retro_full/nwm_retro_v2_XXX.nc*	*nwm_v2_retro_full.ncml*
2.1	https://cida.usgs.gov/thredds/catalog/demo/morethredds/nwm/		
		*v21_reshape/nwm_retro_v2_XXX.nc*	*nwm_v21_retro_full.ncml*

## Technical Validation

Appropriate checks have been made to ensure the correct data are returned from OPeNDAP queries and that nothing was misaligned in the data transformation. Beyond that, the data are exactly as output from the historical simulations.

As a preliminary evaluation of the data quality, we extracted the daily observed streamflow records for the 4,780 USGS NWIS sites from the GAGES-II dataset^25^ with at least 10 years of flow between 1993 and 2018. These records were then compared to the daily mean flows from all three historic versions using the Nash Sutcliffe Efficiency (NSE) metric^52^. NSE provides a normalized goodness-of-fit statistic commonly used in hydrologic model evaluation. However the nature of NSE implies a lower limit of -∞ which can create problems in multi-site model evaluation. As such, Nossent & Bauwens proposed a Normalized NSE (NNSE)^53^ following Eq. 1.

1 $$NNSE=\frac{1}{2-NSE}$$

An NNSE of 1 indicates a “perfect model”, an NNSE = 0.5 indicates the model has the same predictive skill as the mean of the observed time-series, and anything less than 0.5 suggests a user should use the mean of the observed values rather than the model. Although issues of model diagnostics, error evaluation, and interpretation are beyond the scope of this data descriptor, our dataset provides the ability to take a high level look at how model skill evolved spatially from version to version.

**Fig. 1:**
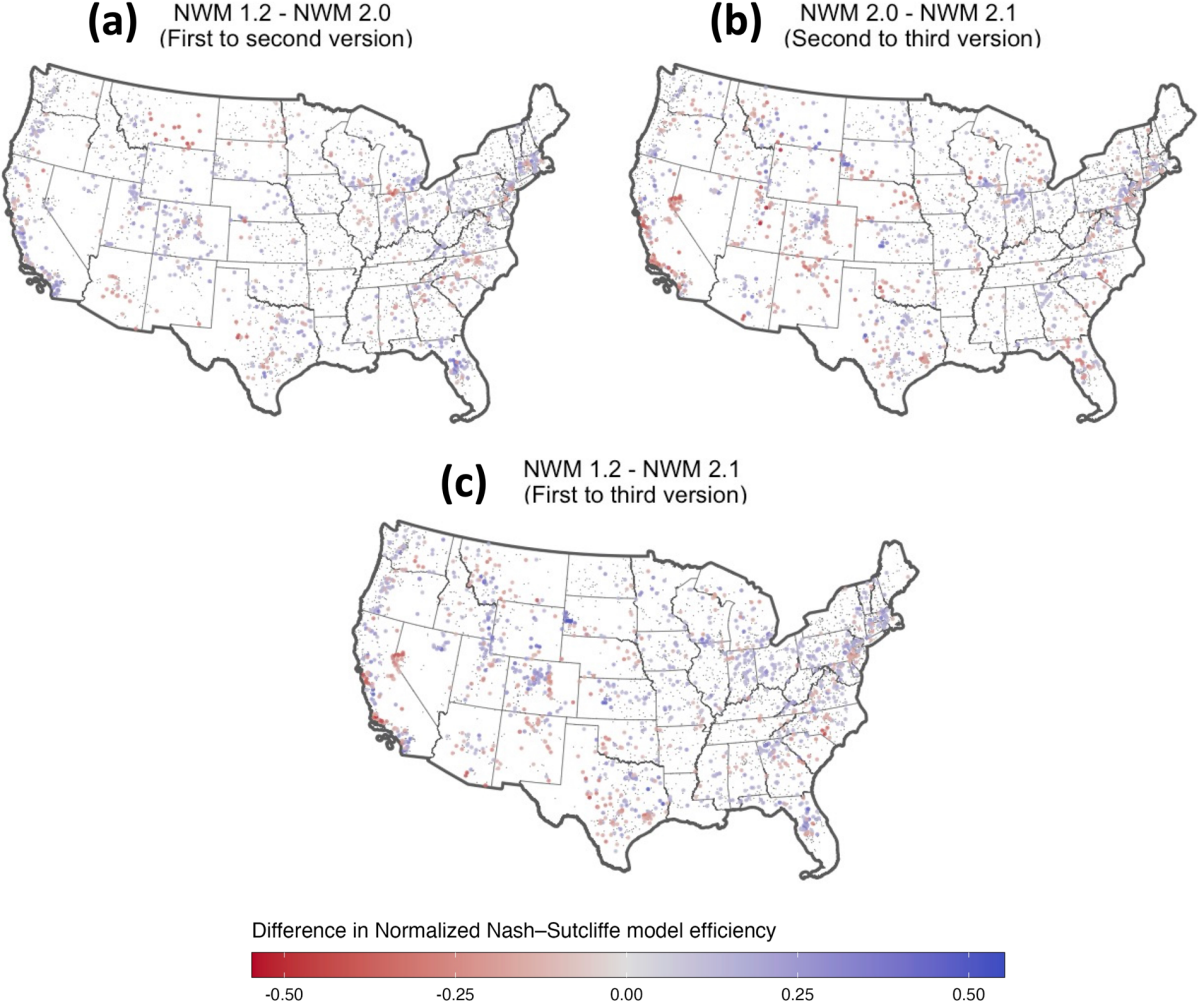
The difference in NNSE between National Water Model versions. Here blue dots indicate an improvement in NNSE at a given site in the new model, and a red location indicates the model degraded in the new version. Panel (**a**) shows the change from version 1.2 to 2.0, panel (**b**) the change from version 2.0 to 2.1, and panel (**c**) the change from version 1.2 to 2.1.

Figure 1 maps the locations where the change in NNSE was larger than ± 0.1 from version to version. Blue locations represent sites that improved with the model advancements, and red locations are those that degraded. This analysis focuses on a single error metric across the entire time series and similar plots focusing on seasonal performance, high and or low flows, or different basin types, may look different.

Starting with panel (1a) we see that version 2.0 increased performance in many areas (specifically the West Coast, the Rocky Mountain Range, Florida and much of the northeastern United States). It was able to do this at the expense of degrading performance in New Mexico, southern Montana, southern Michigan, and areas in the southeastern United States. Overall, 15% of the sites saw NNSE improve by more than 0.1, and 6% degraded by more than 0.1. Looking at panel (1b), we see that version 2.1 improved some of the areas degraded by v2.0 (e.g., southern Montana, southern Michigan, and areas in the southeast) at the expense of degrading performance in coastal California, the Lake Tahoe region, and much of the southwestern United States and the southern Atlantic coast and the eastern seaboard. Overall, version 2.1 saw 13% of the locations increase NNSE by more than 0.1 compared to version 2.0, while 11% of the sites decreased by more than 0.1 NNSE. Lastly, panel (1c) shows the overall improvement across the life of the NWM historic datasets. From version 1.2 to 2.1 the general Rocky Mountains, Midwest, and Florida saw improvement while the Bay Area, Lake Tahoe regions, and parts of the southwestern United States, south Atlantic Coast and  eastern seaboard saw degradation. In total 20% of the sites saw improvement  larger than 0.1 between these models while 10% saw degradation larger than 0.1.

**Fig. 2:**
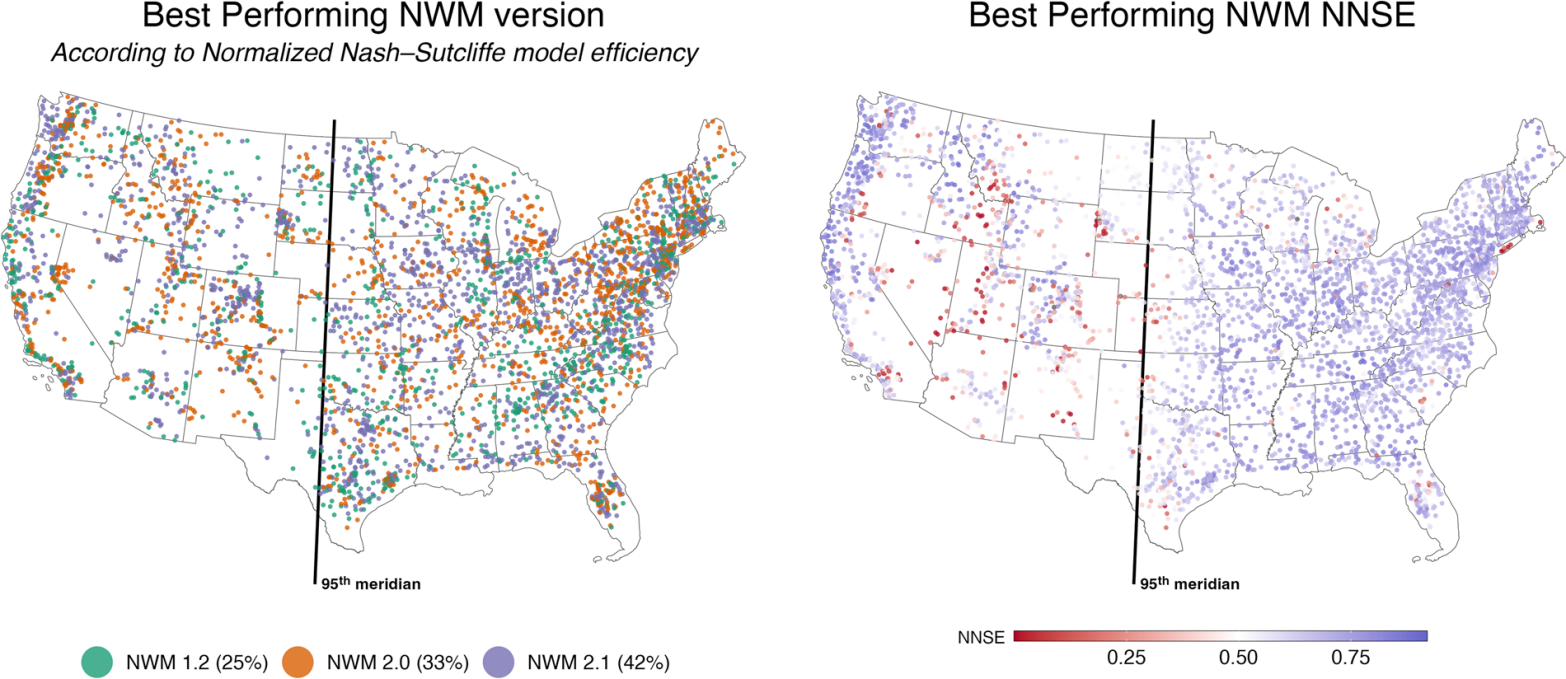
(**a**) Each evaluated NWIS gage is coloured by the best performing NWM historic simulation (**b**) the NNSE of the best performing historic simulation is mapped.

Given the mixed improvement through versions, Fig. 2a colors each location according to the version that performed best. When looking at this map, there are no discernible patterns or clusters indicating one version did better in specific regions. This is notable as a large part of NWM improvement comes from calibration and parameter regionalization^28–30^, and NOAA has acknowledged that improvements in NWM performance, by calibration alone, are beginning to plateau^21^. This realization has spurred the development of the Next Generation Water Modeling Framework (NextGen) that allows for heterogeneous models to be run in different parts of the county within a single platform^21^. Figure 2b looks at the maximum performance that can be achieved if the best version is chosen at each location in a pseudo NextGen representation. In general, the NWM skill is poor west of the 95th meridian (~ central Texas) until the more humid west coast is reached. There are versions that can achieve decent results in the eastern and western slopes of the Rockies, and no models are able to adequately capture the areas surrounding  New York City. When using a combination of all models, 83% of the locations can achieve an NNSE greater than 0.5 compared to 71% in version 1.2, 75% in version 2.0, and 77% in version 2.1.

The takeaway for data users is that decent performance can be seen in most places, however the assumption that the latest model is the best, simply because it’s the most recent, is not accurate. and model choice should depend on the research question and area of study.

Ultimately, this data release describes a new access pattern for the NWM historical streamflow data based on restructuring and serving a time oriented version of the simulated records. The specific data are associated with versions 1.2, 2.0, 2.1 of the NWM but inevitably, a newer version of the NWM will be developed and with it, a new historical product. Although we use this study to provide access to the current simulations, the software code is the reproducible process for formatting the historic product for public consumption and increasing the accessibility that can be used on future NWM historic simulations.

## Usage Notes

Restructuring the historic NWM simulations and serving it through the Consortium of Universities for the Advancement of Hydrologic Science, Inc.'s (CUAHSI) and USGS’s infrastructure allows users to extract a time series for any feature_id of interest without individually managing raw data files. These datasets are open to the public and can be accessed with any DAP enabled software.  

**Fig. 3:**
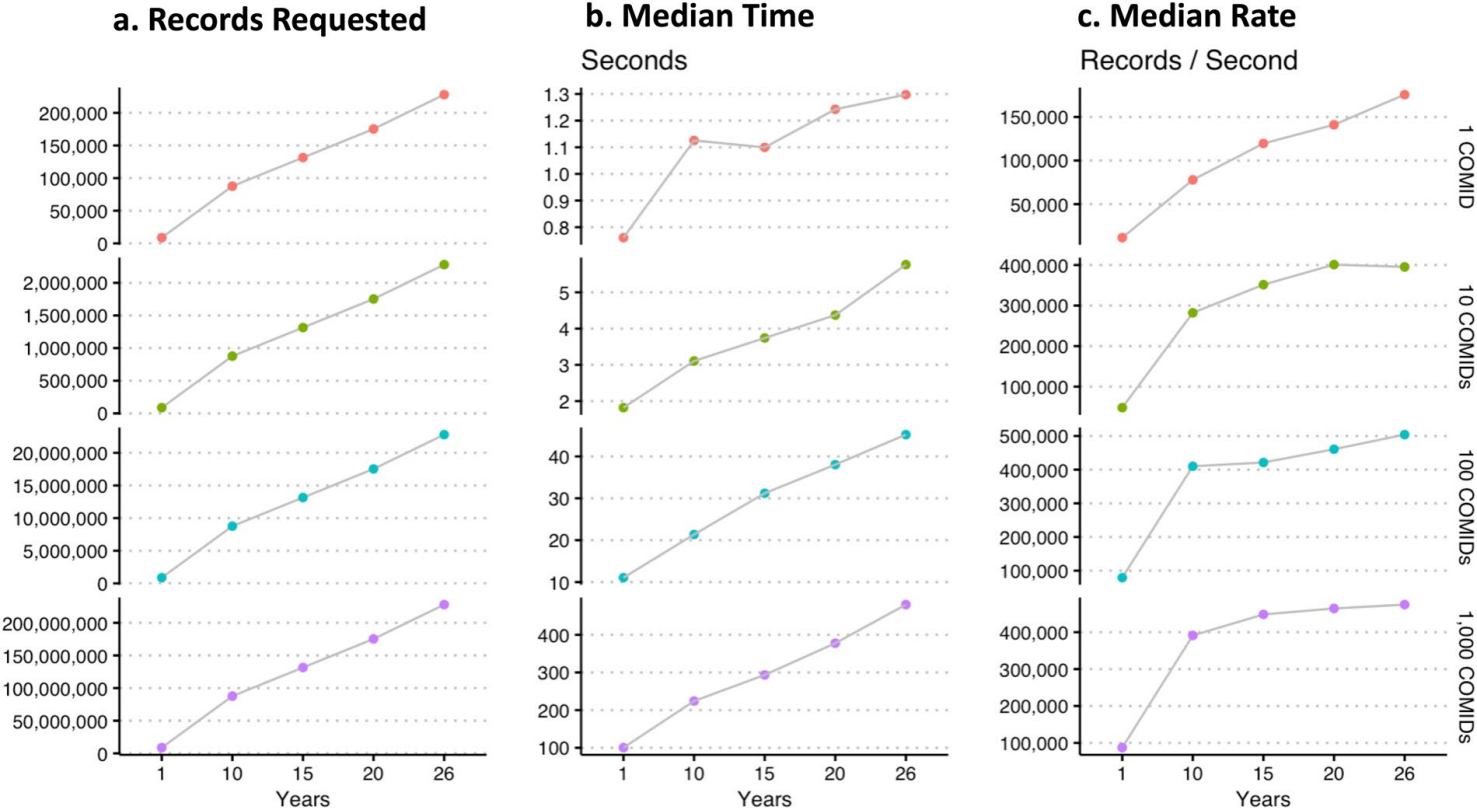
Benchmark performance tests. (**a**) the number of streamflow records requested based on the number of COMID(s) and years requested. (**b**) the median time (seconds) based on the number of COMID(s) and years requested. (**c**) the median extraction rate (records/second) based on the number of COMID(s) and years requested.

With any new web-based dataset, questions about performance and scalability arise. Thus, we benchmark data extraction for 1, 5, 10, 20, and 26 years of hourly data for 1, 10, 100, and 1,000 random feature_ids (these are the same as NHDPlus COMIDs). In total, these requests range from 8,760 to 227,904,000 values (Fig. 3a). Tests were made on a personal laptop with internet speeds approximating 40.8 Mb/s download. Each combination of requests was run five times and the median elapsed time is shown in Fig. 3b. Figure 3c shows the extraction rate of each query (requested records/median elapsed time) which ranged between 2,845 records/second and 515,830 records/second. There is an evident plateau, starting around 400,000 records/second and a maximum performance occurring around 500,000 records/second. Overall, these tests give us confidence the new data sources provide the intended capabilities that allow users to access the historic NWM streamflow dataset with a relatively low barrier to entry and low computational requirements.

Building OPeNDAP queries is not a trivial task, and identifying the appropriate feature_id and time  ID, and its positions in the aggregated resource is a repetitive process prone to error. To further improve accessibility, we offer the *nwmTools* R package^54^ which uses the RNetCDF^55^ NetCDF client for sending/retrieving OPeNDAP requests. The primary package function for historical data is *readNWMdata(…)*, which intentionally mirrors functionality provided in the USGS *dataRetrieval* package^44^.

By default, the function returns all hourly data from version 2.1 in UTC for a user supplied area of interest (AOI), COMID(s), or USGS streamgage ID(s). Additional parameters allow users to narrow the start and end date, adjust the time zone, and specify the model version desired. The package also provides a family of functions for aggregating streamflow records to other time chunks (e.g., monthly, water year, or season) and for appending the spatial NHDPlus features to the output data.frame (based on webservices rather than the preceeding the Esri geodatabase). Here it should be noted that data can only be returned for NHD features included in the NWM (e.g., the model does not include NHD flowlines associated with waterbodies and thus only NA values would be returned).

**Fig. 4:**
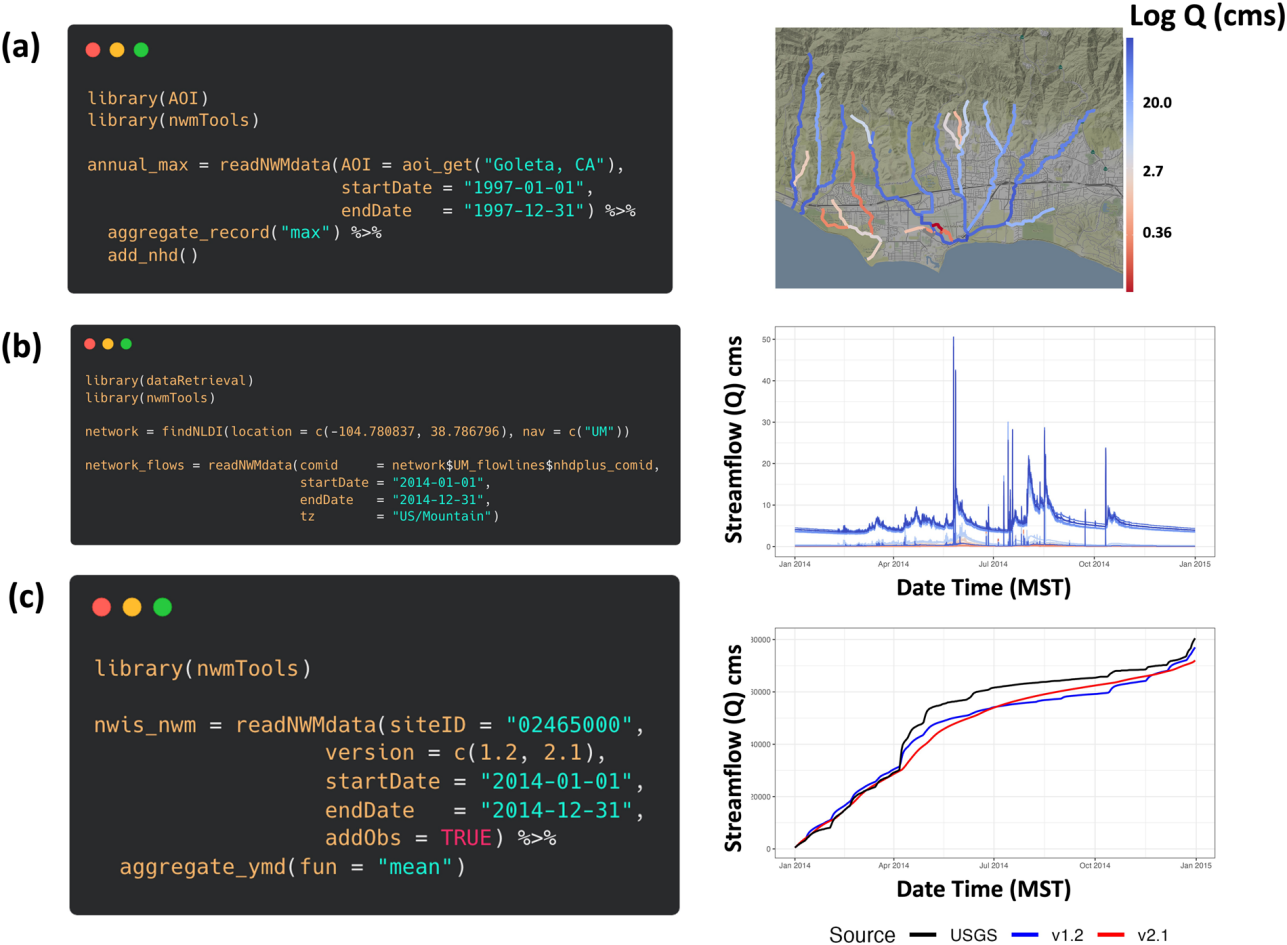
(**a**) Maximum annual NWM streamflows for the areas of Goleta, California found by integrating *AOI* and *nwmTools*. The data are plotted on the spatial NHDPlus flowlines (**b**) Annual hourly flows for the complete upstream mainstem of a defined point found by integrating *dataRetrieval* and *nwmTools*. The data for all reaches are plotted with red colors near the headwaters graduating to blue as they reach the outlet (**c**) Multi-versions of the NWM compared to observations at a gaged location using *nwmTools*. Cumulative flow plots are shown to see the difference in records.

To highlight these tools, we present three use cases (Fig. 4) that show how to find NWM data by AOI, by COMID, and by USGS streamgage number. Indirectly we show ways for finding this information using open-source packages.

The first use case (4a) illustrates how the *nwmTools* can find NWM data for a given area and time range (*readNWMdata*), summarize the hourly data to an annual mean (*aggregate_record*), and append NHDPlus geometries to the forecast (*add_nhd*). A key feature of the NHD data model is the ability to traverse the hydrographic flow network and modern data systems like the Network Linked Data Index (NLDI) are capitalizing on the graph nature of the hydrographic networks to facilitate feature discovery and indexing^56,57^. The NLDI has multiple programmatic interfaces available in R (dataRetrieval^44^, nhdplusTools^58^) and Python (HyRiver)^59^ that are supported by the web framework developed as part of the Open Water Data Initiative^56,60^. Panel 4b illustrates a use case to find the annual flow records upstream of a known location in Colorado Springs, Colorado. Starting from this point, the NLDI can return the COMIDs associated with the upstream mainstem (UM) which can be passed directly to *readNWMdata* to get all flow records for 2014 in Mountain Standard Time zone. These time series are colored using a red to blue palette where the darkest reds are the headwater reaches and the darkest blue is the outlet reach. Lastly, many efforts aimed at model evaluation, calibration, and improvement need to identify simulation records that match some gaged record. We can use a USGS site number to query the historic NWM simulation *and* the USGS observations. Panel 4c shows how to find two versions of the NWM and the observed record at USGS site number 02465000. The data are returned in a way that is directly comparable, for example, as a cumulative flow plot.

In all three examples the underlying data records are what allow these discovery workflows to succeed. To this end, these use cases could prompt the consideration of distributing the operational products in a similar way which would allow any variation of the workflows illustrated here to be used with the operational forecasts.

## Data Availability

All code for data download and reformatting can be found in the appropriate USGS repository^50^. The *nwmTools* R package is available on GitHub and the dataset is documented and published via HydroShare^51^. All the data are currently open, and publicly available at this URL: https://www.hydroshare.org/resource/84c2b029f97343a59d0739115d4087f1/.
