# Publisher Correction: Restructuring and serving web-accessible streamflow data from the NOAA National Water Model historic simulations

**DOI:** 10.1038/s41597-024-03035-3

**Published:** 2024-02-12

**Authors:** J. Michael Johnson, David L. Blodgett, Keith C. Clarke, Jon Pollak

**Affiliations:** 1 Lynker, Fort Collins, CO USA; 2 University of California, Santa Barbara, USA; 3 U.S. Geological Survey, Reston, USA; 4 Consortium of Universities for the Advancement of Hydrologic Science, Inc, Cambridge, USA

**Keywords:** Hydrology, Environmental impact

Correction to: *Scientific Data* 10.1038/s41597-023-02316-7, published online 20 October 2023

The Figure 1 image was incorrectly listed as Figure 2, and vice versa, in the original version. This has now been corrected in the pdf and HTML versions of the paper, alongside some typographical mistakes.

